# Supplementary material for: Unveiling Non-Covalent Interactions in Novel Cooperative Photoredox Systems for Efficient Alkene Oxidation in Water
Source: Molecules. 2024 May 18;29(10):2378. doi: 10.3390/molecules29102378 (PMC11123843; doi:10.3390/molecules29102378)
Supplement: Supplementary file 1 [file molecules-29-02378-s001.zip › molecules-3004003-supplementary.pdf]

SUPPORTING INFORMATION

Unveiling Non-Covalent Interactions in Novel Cooperative Photoredox Systems for Efficient Alkene Oxidation in Water

Isabel Guerrero,[a] Clara Viñas,[a] Francesc Teixidor [a] and Isabel Romero,[b]**

[a]Institut de Ciencia de Materials de Barcelona, ICMAB-CSIC, Campus UAB, E-08193 Bellaterra, Spain. and Serveis Tècnics de Recerca, Universitat de Girona, C/ M. Aurèlia Campmany, 69, E-17003 Girona, Spain.

[b]Departament de Química and Serveis Tècnics de Recerca, Universitat de Girona, C/ M. Aurèlia Campmany, 69, E-17003 Girona, Spain.

*Correspondence to: marisa.romero@udg.edu; teixidor@icmab.es;

TABLE OF CONTENTS

Figure S1. IR spectrum of **Ag[Cl$_6$-1].**

Figure S2. IR spectrum of **5.**

Figure S3. a) $^1H\{^{11}B\}$-NMR and b) $^{11}B\{^1H\}$-NMR spectra of **Ag[Cl$_6$-1]** compound in acetone-d$_6$.

Figure S4. a) ^1H-NMR; b) $^1H\{^{11}B\}$-NMR; c) $^{11}B\{^1H\}$-NMR d) ^{11}B-NMR and e) COSY NMR spectra of **5.** compound in acetone-d$_6$.

Figure S5. UV-visible of **Ag[Cl$_6$-1]** compound in CH_2Cl_2.

Figure S6. CV of a) **Ag[Cl$_6$-1]** compound in CH_3CN + 0.1 M TBAH vs Ag/AgCl; b) **5** in CH_3CN + 0.1 M TBAH vs Ag/AgCl; and c) **5** in a phosphate buffer (pH = 7.12) vs Ag/AgCl; scan rate V= 100 mV/s.

Figure S7. Plot of conversion as a function of time for the photoredox catalysis of styrene. Conditions: **4** (0.01 mM), styrene (20 mM), $Na_2S_2O_8$ (26 mM), 5 ml aqueous solution at pH=7, light irradiation (2.2 W, λ~300 nm).

Figure S8. ESI-MS spectra of **5**.

Table S1. Photooxidation tests performed with complex **4**. Conditions: **4** (0.01 mM), substrate (20 mM), $Na_2S_2O_8$ (26 mM), 5 mL aqueous solution at pH=7.

Table S2. Photooxidation of epoxides performed with complex **4**. Conditions: **4** (0.01 mM), epoxide (20 mM), $Na_2S_2O_8$ (26 mM), 5 mL aqueous solution at pH=7.

Table S3. Photooxidation tests performed with **5** complex. Conditions: **5** (0.001 mM), substrate (20 mM), $Na_2S_2O_8$ (26 mM), 5 mL aqueous solution at pH=7.

Figure S1. IR spectrum of **Ag[Cl$_6$-1].**

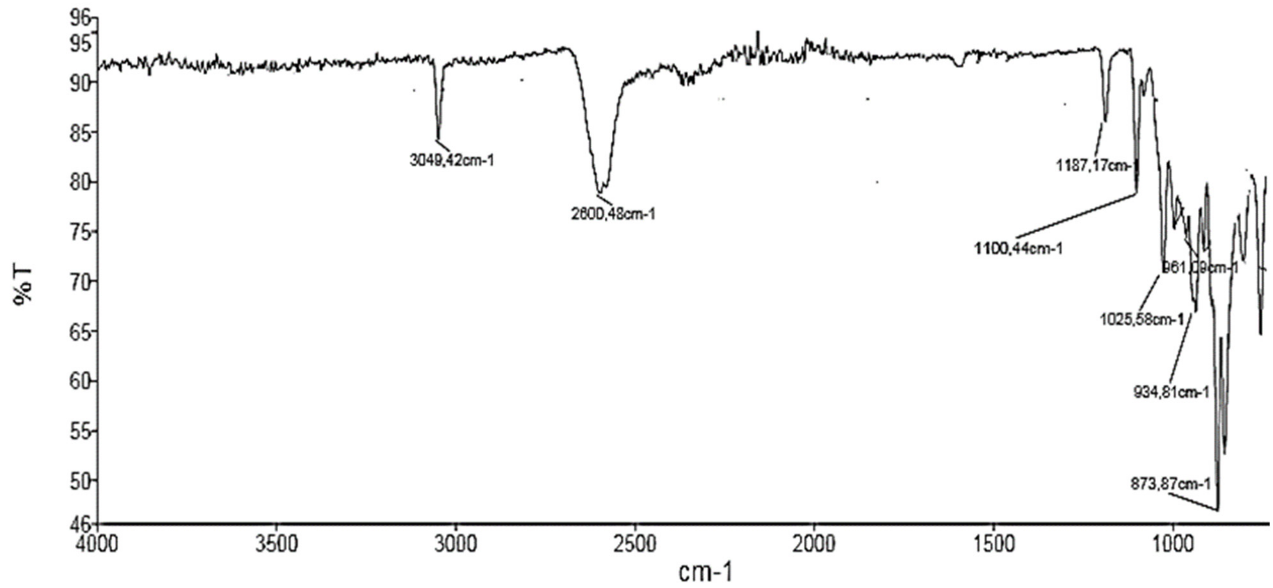

Figure S2. IR spectrum of **5**.

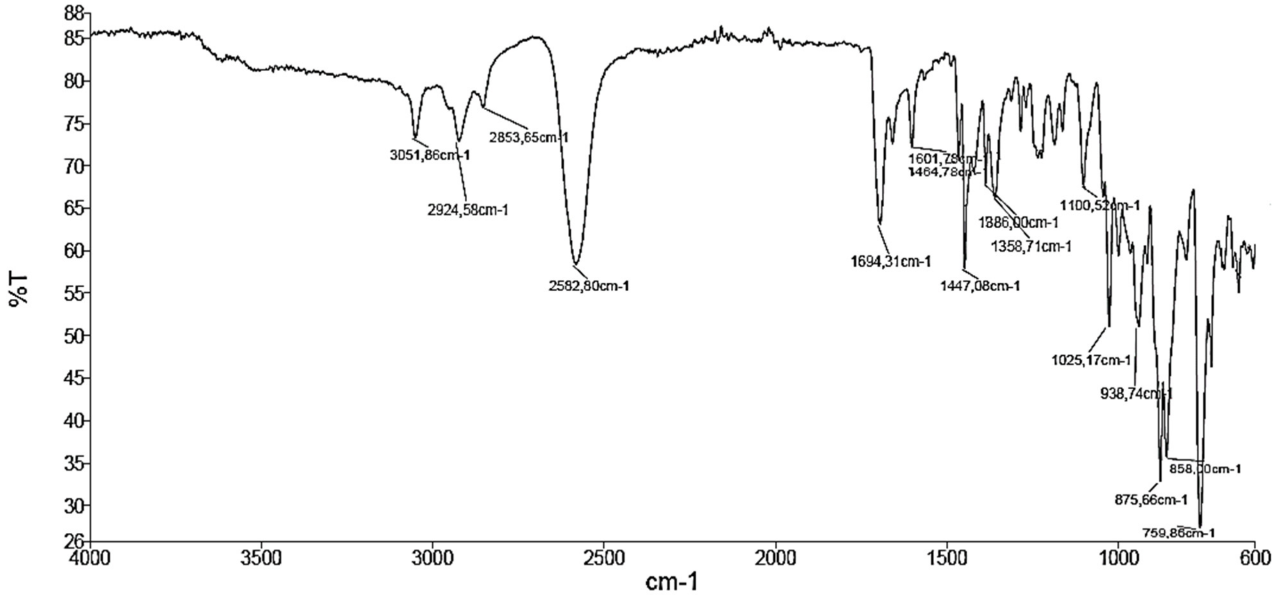

Figure S3. a) $^{1}H\{^{11}B\}$-NMR and b) $^{11}B\{^{1}H\}$-NMR spectra of **Ag[Cl$_6$-1]** compound in acetone-d$_6$.

a)

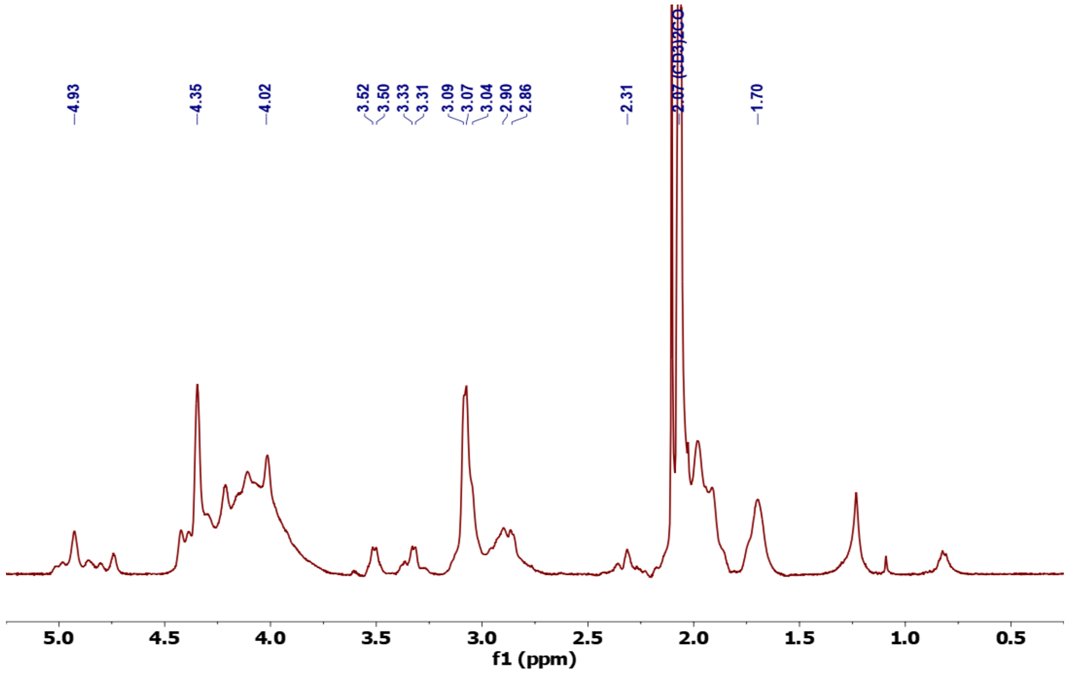

b)

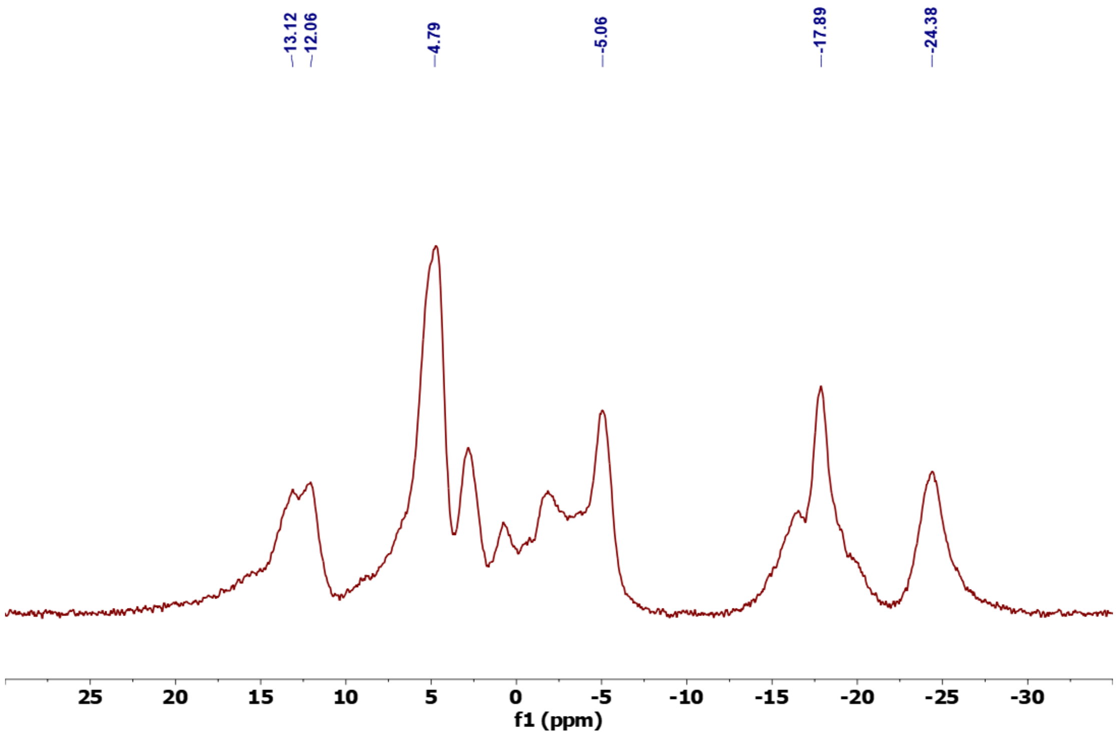

Figure S4. a) ^{1}H-NMR; b) $^{1}H\{^{11}B\}$-NMR; c) $^{11}B\{^{1}H\}$-NMR d) ^{11}B-NMR and e) COSY NMR spectra of **5.** compound in acetone-d6.

a)

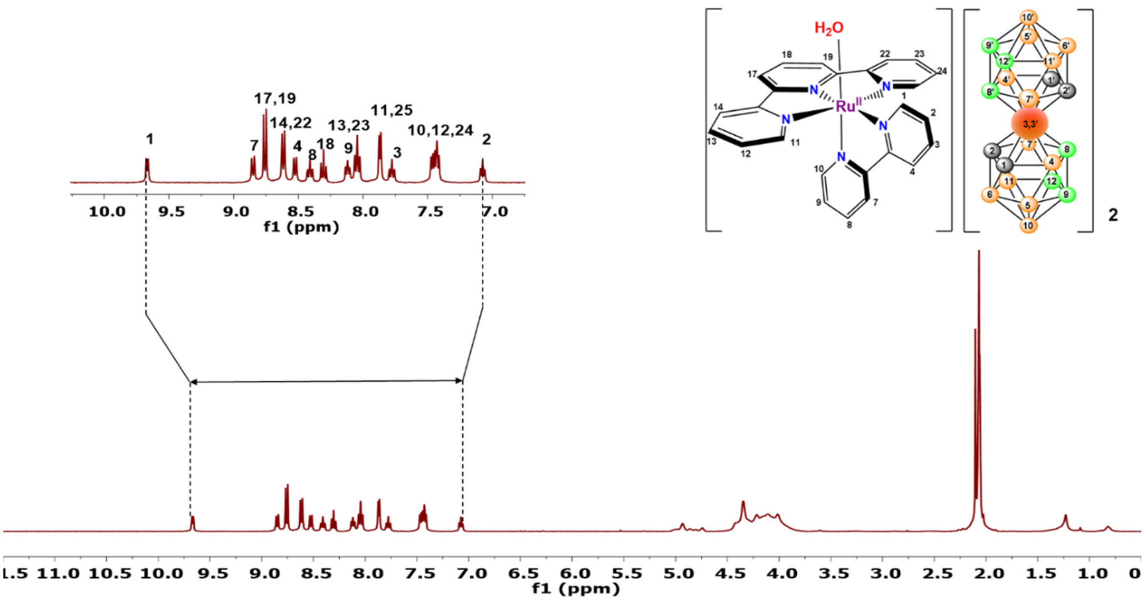

b)

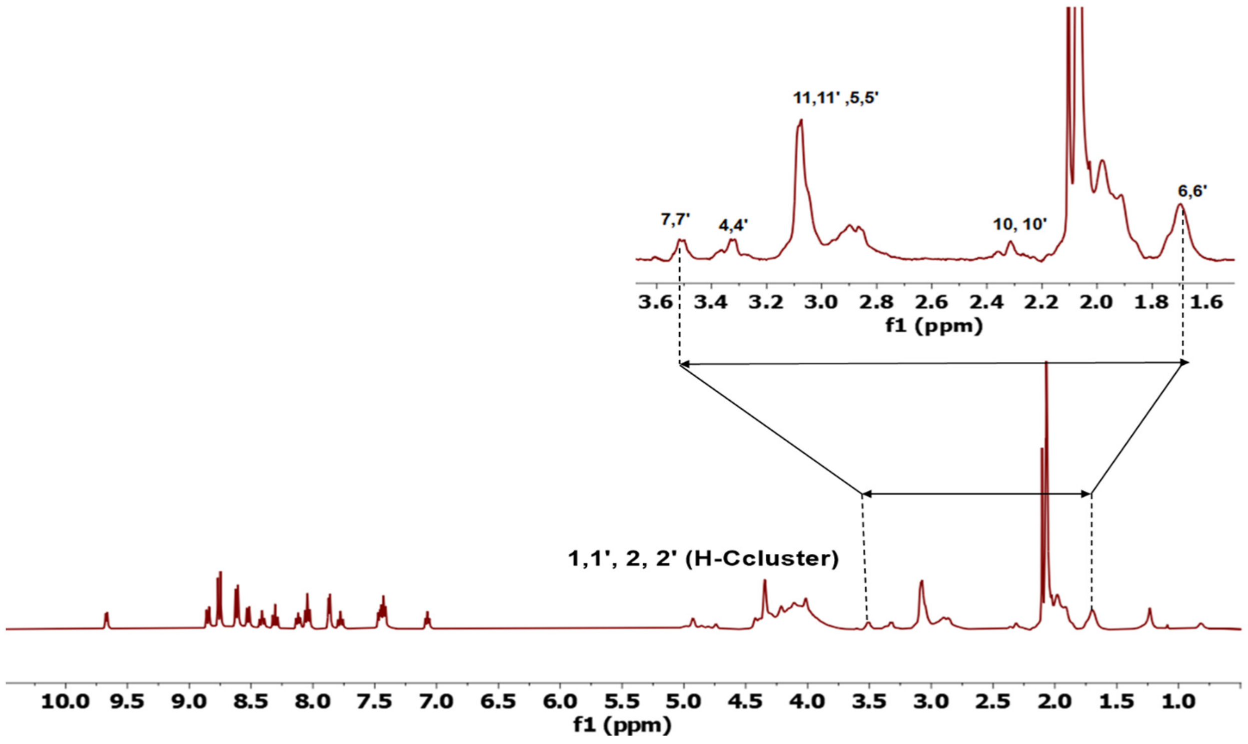

c)

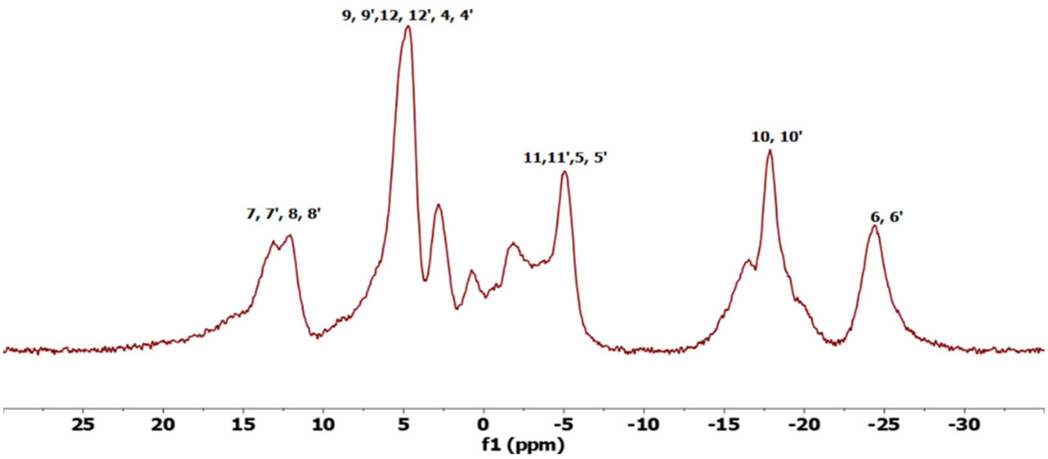

9, 9',12, 12', 4, 4'
11,11',5, 5'
10, 10'
7, 7', 8, 8'
6, 6'
25
20
15
10
5
0
-5
-10
-15
-20
-25
-30
f1 (ppm)

d)

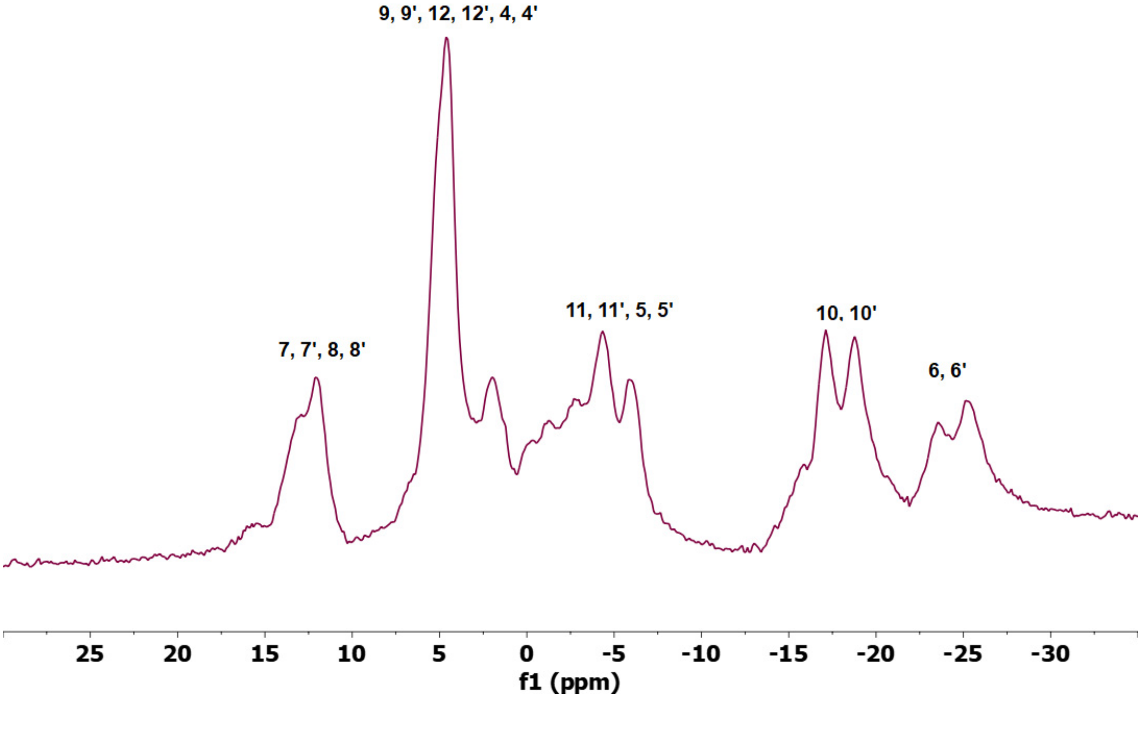

9, 9', 12, 12', 4, 4'
11, 11', 5, 5'
10, 10'
7, 7', 8, 8'
6, 6'
25
20
15
10
5
0
-5
-10
-15
-20
-25
-30
f1 (ppm)

e)

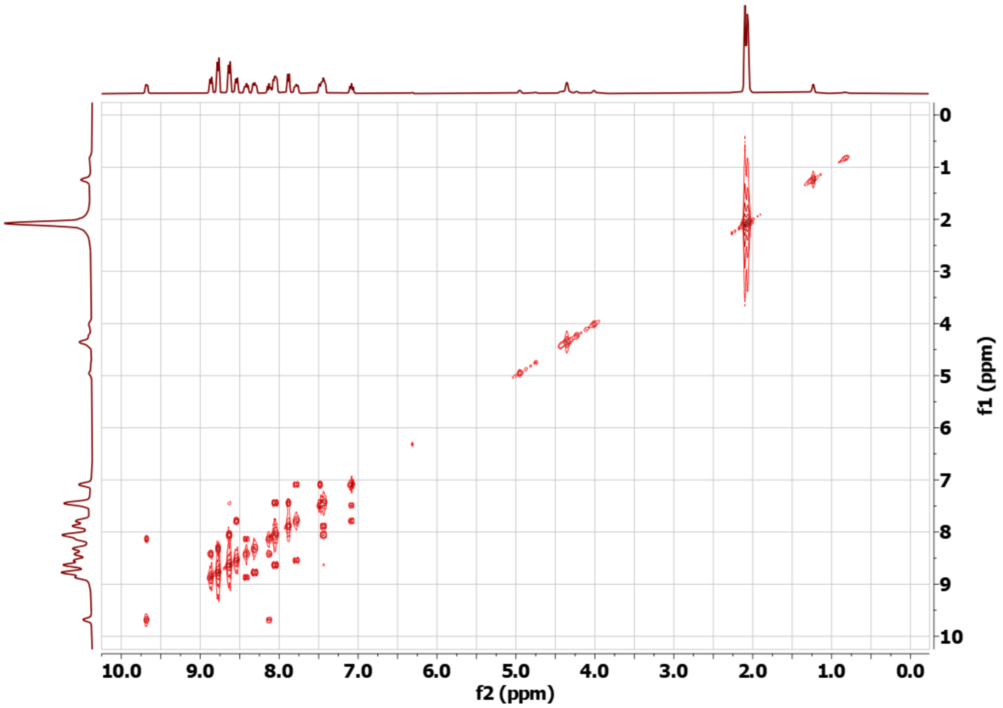

Figure S5. UV-visible of **Ag[Cl$_6$-1]** compound in CH_2Cl_2.

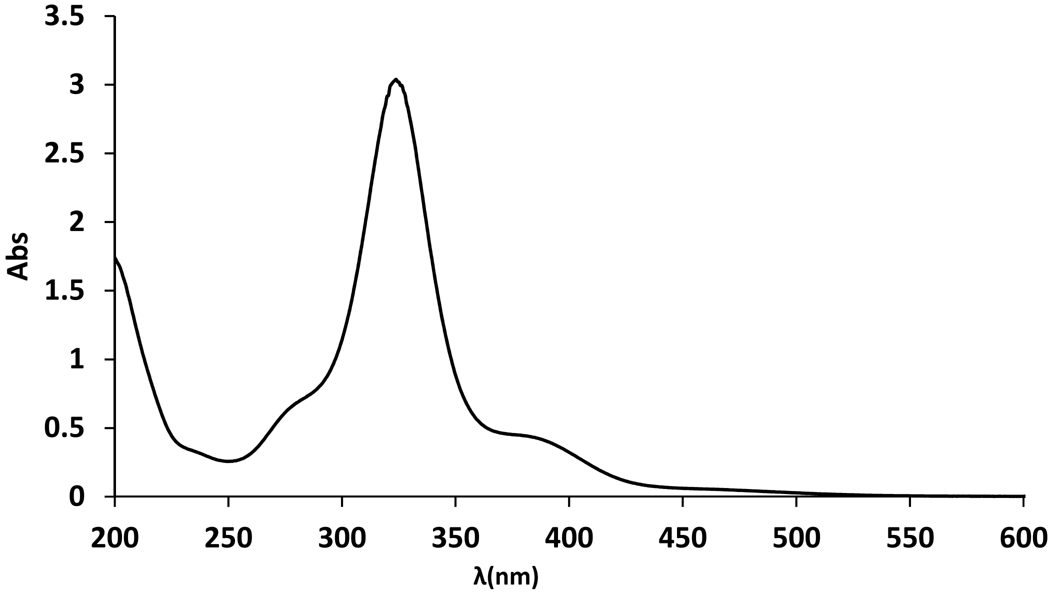

Figure S6. CV of a) **Ag[Cl$_6$-1]** compound in CH_3CN + 0.1 M TBAH vs Ag/AgCl; b) **5** in CH_3CN + 0.1 M TBAH vs Ag/AgCl; and c) **5** in a phosphate buffer (pH = 7.12) vs Ag/AgCl; scan rate V= 100 mV/s.

a)

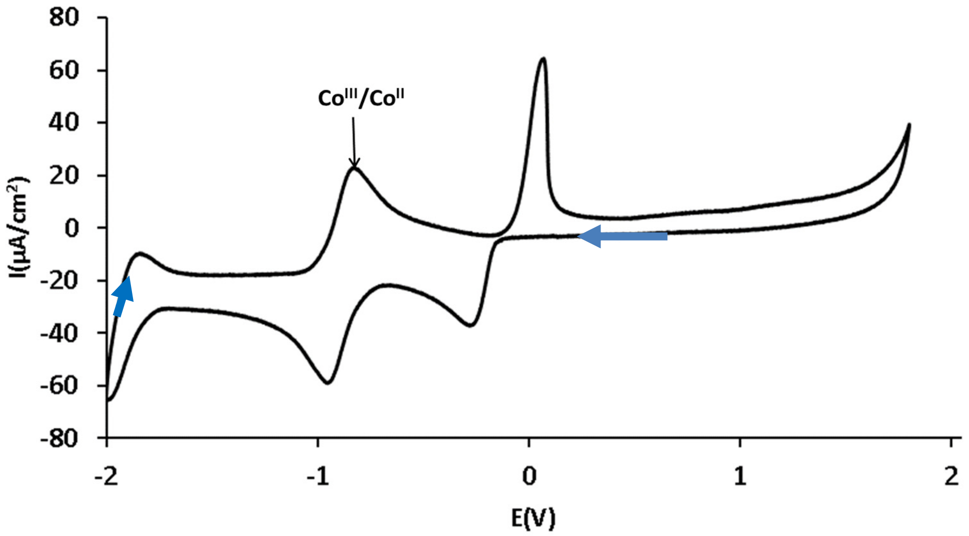

b)

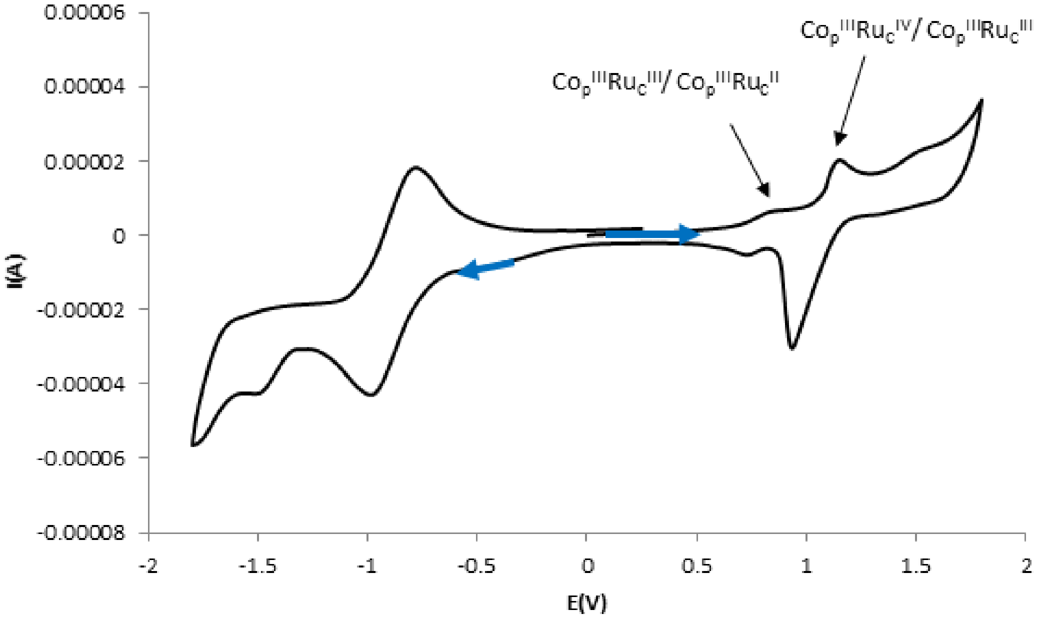

c)

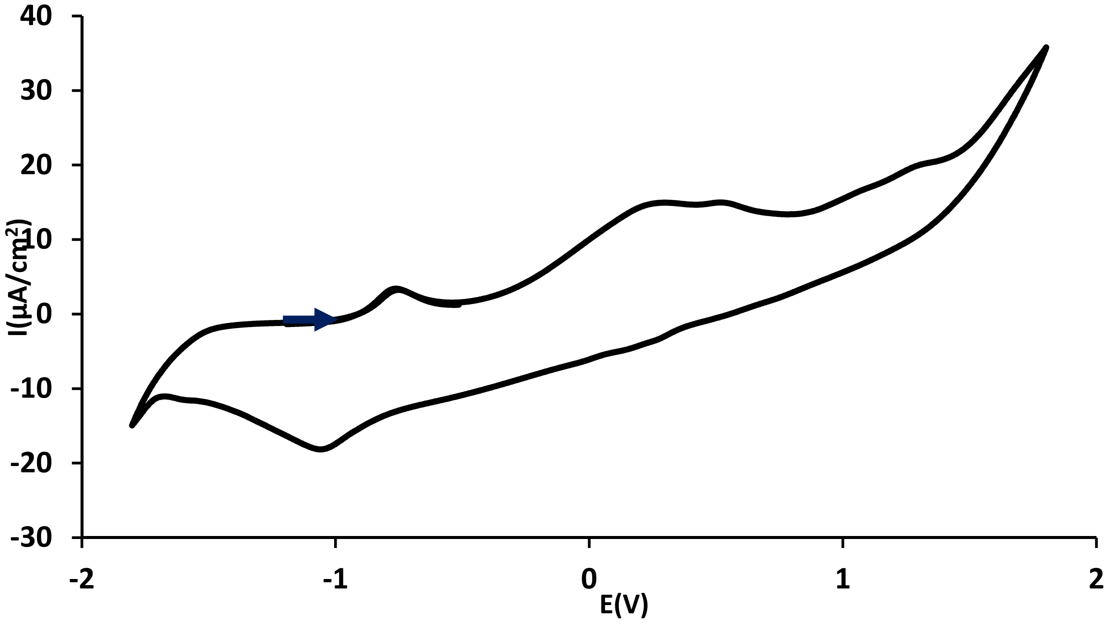

Figure S7. Plot of conversion as a function of time for the photoredox catalysis of styrene. Conditions: **4** (0.01 mM), styrene (20 mM), $Na_2S_2O_8$ (26 mM), 5 ml aqueous solution at pH=7, light irradiation (2.2 W, λ ~300 nm).

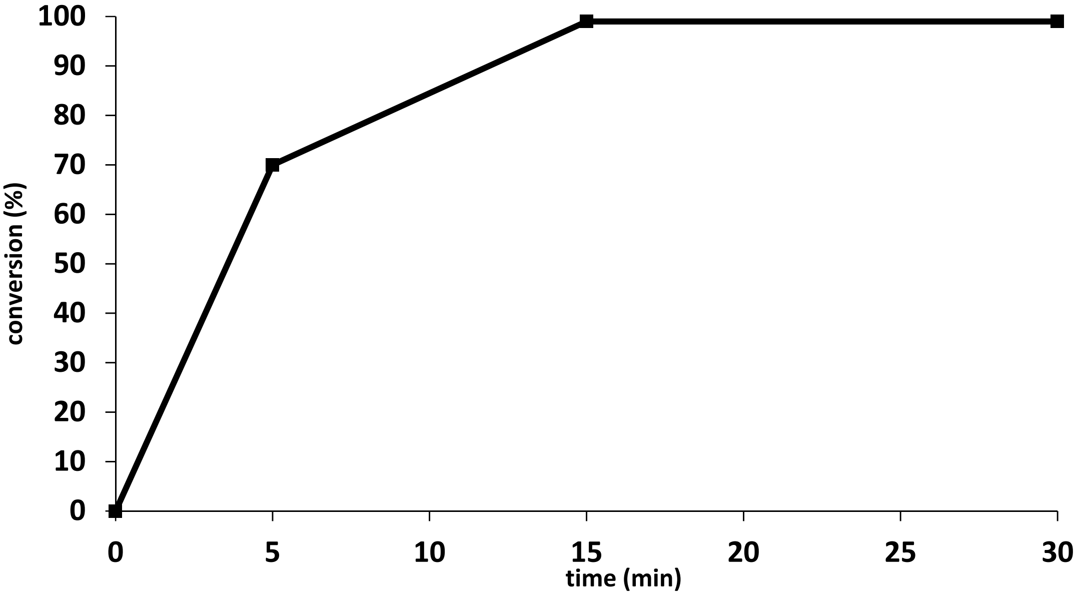

Figure S8. ESI-MS spectra of **5**.

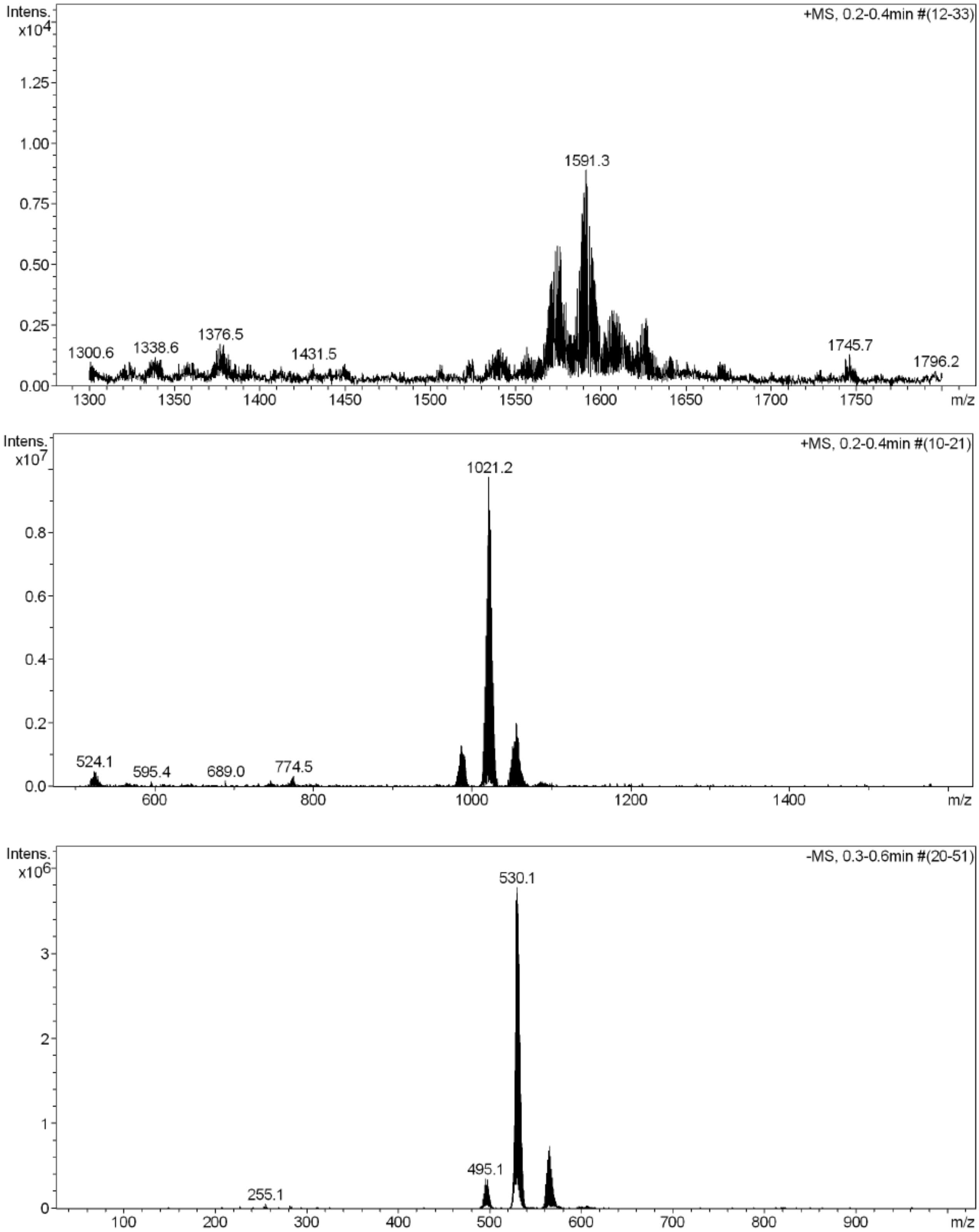

Table S1. Photooxidation tests performed with **4** complex.

Entry	substrate	Conv.%		Yield.(selec.)%		Yield(selec.)%	
1	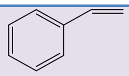	70 [a]1<br>≥99[b]1	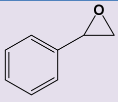	67(96)[a]<br>57(57)[b]	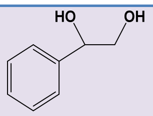	1(1)[a]<br>35(35)[b]	
2	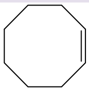	≥99[b]<br>≥99[c]	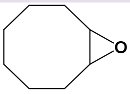	67(67)[b]<br>55(55)[c]	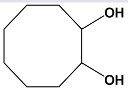	33(33)[b]<br>45(43)[c]	
3	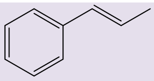	96[b]<br>≥99[c]	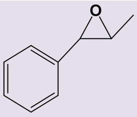	76(79)[b]<br>69(69)[c]	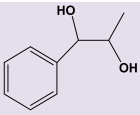	20(21)[b]<br>31(31)[c]	
4	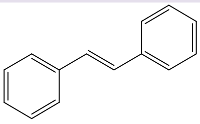	89[b]<br>97[c]	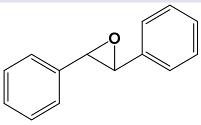	85(96)[b]<br>52(54)[c]	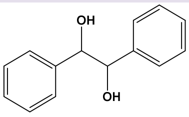	4(5)[b]<br>45(46)[c]	
5	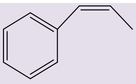	≥99[b]<br>≥99 [c]	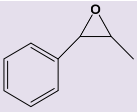	91[59/32, cis/trans](91[2])[b]<br>60[36/24, cis/trans](60[2])[c]	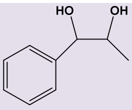	9(9)[b]<br>40(40)[c]	
7	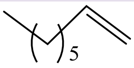	≥99[b]<br>≥99 [c]	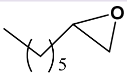	65(65)[b]<br>39(39)[c]	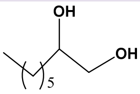	15(15)[b]<br>24(24)[c]	Other products<br>20[4] (20)[b]<br>37[4] (37)[c]

Conditions: **4** (0.01 mM), substrate (20 mM), $Na_2S_2O_8$ (26 mM), 5 mL aqueous solution at pH=7. [a] 5 min of reaction [b] 15 min of reaction. [c] 30 min of reaction.[1] benzaldehyde and benzoic acid produced as another byproducts. [2]selectivity with respect the overall epoxide produced.[3]yield with diol and benzoic acid from vinyl produced. [4]octanal and octanoic produced.

Table S2. Photooxidation of epoxides performed with complex **4**.

Entry	substrate	Conv.%	Product	Yield(selec.)%
1	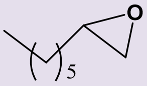	≥99[a]<br>≥99[b]	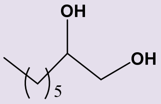	90(90)[a]<br>60(60)[b]
2	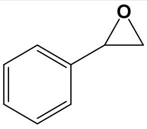	≥99[a]<br>≥99[b]	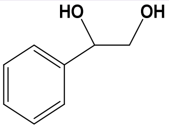	55(55)[b]<br>65(65)*[c]
3	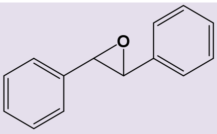	97[a]<br>≥99[b]	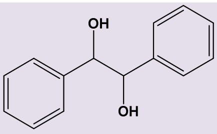	97(≥99)[b]<br>≥99(≥99)[c]

Conditions: **4** (0.01 mM), substrate (20 mM), $Na_2S_2O_8$ (26 mM), 5 mL aqueous solution at pH=7. [a]15 min of reaction [b]30 min of reaction. Yield and selectivity with respect the overall diol produced.* [[yield (selectivity)] with respect the obtention of benzaldehyde and benzoic acid.

Table S3. Photooxidation tests performed with **5** complex.

Entry	substrate	Conv.%		Yield.(selec.)%		Yield(selec.)%
**1**	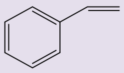	75 [a]1	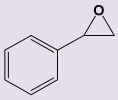	23(31)[a]	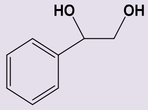	38(51)[a]
**2**	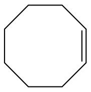	92[a]<br>96[b]	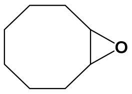	92(>99)[a]<br>53(55)[b]	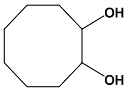	43(45)[b]
**3**	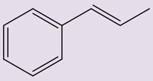	87[b]	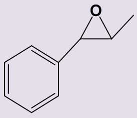	87(>99)[b]		
**4**	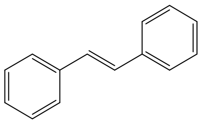	>99[a]<br>>99b]	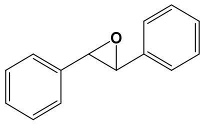	86(86)[a]<br>83(83)[b]	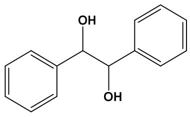	14(14)[a]<br>17(17)b]
**5**	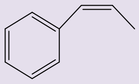	88[a]<br>89 [b]	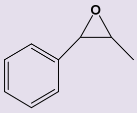	50[50, trans](57)[a]<br>50[51, trans](57)b]	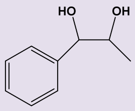	38(43)[a]<br>37 (42)[b]

Conditions: **5** (0.001 mM), substrate (20 mM), $Na_2S_2O_8$ (26 mM), 5 mL aqueous solution at pH=7. [a] 15 min of reaction. [b] 30 min of reaction. [1] benzaldehyde and benzoic acid produced as another byproducts.
